# Long-term mortality in patients with end-stage renal disease undergoing hemodialysis and peritoneal dialysis: a propensity score matching retrospective study

**DOI:** 10.1080/0886022X.2024.2321320

**Published:** 2024-03-14

**Authors:** Pengjie Zhang, Liru Xun, Nan Bao, Ding Tong, Bin Duan, Du Peng

**Affiliations:** Department of Nephrology, Shaanxi Provincial People’s hospital, Xian Jiaotong University, Xian, China

**Keywords:** End-stage renal disease, hemodialysis, peritoneal dialysis, survival rate, influencing factors

## Abstract

**Background:**

Hemodialysis (HD) and peritoneal dialysis (PD) are effective ways to treat end-stage renal disease (ERSD). This study aimed to investigate the differences in survival and the factors that influence it in patients with end-stage renal disease treated with HD or PD.

**Methods:**

We retrospectively analyzed factors related to all-cause death with renal replacement therapy and compared the long-term mortality between HD and PD strategies in patients with ESRD who started HD or PD treatment in our renal HD center between January 1, 2008, and December 1, 2021.

**Results:**

Overall, 1,319 patients were included, comprising 690 and 629 patients in the HD and PD groups, respectively, according to the inclusion criteria. After propensity matching, 922 patients remained, with 461 (50%) patients each in the two groups. There were no significant differences in the 1-, 2-, 3-, and 4-year mortality rates between the HD and PD groups (all *p* > .05). However, the 5- and 10-year mortality rates of the matched patients were 15.8%. 17.6% in the HD group and 21.0%. 27.3% in the PD group, respectively. The 5- and 10-year mortality rates were significantly lower in the HD group (all *p* < .05) as compared to the PD group. After matching, Kaplan–Meier curve analysis with log-rank test was performed, which showed a significant difference in the survival rates between the two groups (*p* = .001). Logistic multifactor regression analysis revealed that age, weight, hypertension, serum creatinine, and combined neoplasms influenced the survival rate of patients with ESRD (*p* < .05). In contrast, age, hypertension, parathyroid hormone (PTH), serum creatinine, and peripheral vascular diseases (PVD) influenced the survival rate of patients in the HD group (*p* < .05), and age and weight influenced the survival rate of patients in the PD group (*p* < .05).

**Conclusions:**

This study found that long-term mortality rates were higher in the PD group than that in the HD group, indicating that HD may be superior to PD.

## Introduction

End-stage renal disease (ESRD) refers to the end-stage of chronic renal insufficiency caused by multiple reasons, whereby patients’ renal function gradually declines and toxins and body fluids accumulate in the body, causing significant damage to several systems in the body. Furthermore, the severity of the disease can have a severe impact on patient’s quality of life and safety.^1^

Hemodialysis (HD) and peritoneal dialysis (PD) are effective methods to treat ESRD and can significantly improve patient survival rates. However, studies have shown that 13.2% of patients undergoing maintenance dialysis die each year, especially older adults (>70 years). Moreover, the 5-year survival rate is even lower than 40%.^2^^,^^3^

At the same time, the survival rates of patients undergoing HD and PD are inconsistent across different studies. Weinhandl et al. studied 6,337 patients undergoing HD and PD for 4 years and found that PD had a low mortality rate in the first few months; however, with an increase in the duration for which PD was administered, the survival rate was affected by insufficient nutrition intake and gradually emerging hypoproteinemia. At 2 years, the survival rate of patients undergoing HD was significantly improved than that of patients undergoing PD (71.1% vs. 68.0%, *p* < .01), which was more evident in patients with cardiovascular disease and diabetes. There was no significant difference in the survival rates between the two treatment methods after 4 years of follow-up (48.4% vs. 47.3%, *p* = .50).^4^ Zang et al. followed studied 309 patients undergoing PD and 233 patients undergoing on HD for 3 years and found that the 3-year survival rate of patients on HD was only 57.8%, and that of patients on PD was 64.8%. It has been reported that PD is superior to HD and that HD, aging, and hypokalemia are independent factors that affect the survival rate of patients undergoing dialysis.^5^

The choice of PD and HD modalities can affect national policymaking, treatment expenditure, patient quality, and survival rate. Regarding the advantages and disadvantages of dialysis modalities for patients with ESRD, the varying results of different studies may be related to the differences in region, race, medical conditions, and treatment regimens, among other factors.

With the development of health insurance policies and dialysis technology, the number of patients receiving dialysis has expanded considerably. However, there are few determinants of all-cause death in patients with ESRD receiving renal replacement therapy in China, and there has been no comparison of long-term survival between the two groups.

In this study, we retrospectively analyzed the survival of patients with ESRD who started HD or PD treatment at our renal HD center between January 1, 2008, and December 1, 2021. The present study aimed to report the following: (i) factors related to all-cause death in patients with ESRD undergoing renal replacement therapy, and (ii) compare the long-term mortality between HD and PD strategies in patients with ESRD.

## Methods

### Data collection

This study included patients diagnosed with ESRD who received HD or PD treatment at our center from January 1, 2008, to December 31, 2021. The main inclusion criteria for the final analysis were as follows: (1) sufficient clinical data and laboratory examinations and (2) successful follow-up until the endpoint event.

Patients with missing data were excluded from the analysis. The key exclusion criteria were as follows: (1) recent acute coronary syndrome, (2) acute kidney injury, (3) poor control of blood pressure and glucose, (4) serious or critical condition, (5) transfer to other center sand (6) conversion from HD to PD or from PD to HD. The data elements and definitions of each variable were in accordance with the kidney disease: Improving Global Outcomes 2012 Clinical Practice Guideline.^6^ The study was approved by the Human Research Ethics Committee of Shaanxi Provincial Hospital and was conducted in accordance with the ethical standards of the institutional and/or national research committee and the 1964 Helsinki Declaration and its later amendments or comparable ethical standards. Written informed consent was obtained from all the patients.

### Study design

The clinical data of all admitted participants were exported from electronic medical records, including ethnicity, sex, age, weight, urine output, body mass index (BMI), primary disease, hemoglobin, albumin, N-terminal pro-brain natriuretic peptide (NT-proBNP), parathyroid hormone (PTH), residual renal function, blood creatinine, blood urea nitrogen, and the presence of combined coronary artery disease, heart failure, PVD, diabetes mellitus, hypertension, malignant neoplastic disease, chronic obstructive pulmonary disease(COPD), and myocardial infarct(MI). The enrolled patients were divided into PD and HD groups based on the dialysis modality used.

### Follow-up and the endpoint event

All admitted participants’ retrospective follow-up through outpatient visiting, rehospitalization, and telephone commenced on the day of enrollment and ended at the first occurrence of the endpoint event or in December 2021. The endpoint event was defined as an all-cause death, which refers to death from any cause that occurred during the follow-up period.

### Propensity score matching and covariates

We performed a propensity score-matched Cox proportional hazard analysis. Propensity score matching (1:1) was performed based on the nearest neighbor. A caliper of 0.2 was used to adjust the measured confounders between the HD and PD groups at baseline before comparison. Propensity scores were calculated considering the following baseline variables as covariates: ethnicity, sex, age, weight, urine volume, BMI, primary disease, hemoglobin, albumin, NT-proBNP, PTH, residual renal function, blood creatinine, blood urea nitrogen, presence of combined coronary heart disease, heart failure, PVD, diabetes mellitus, hypertension, malignant neoplastic disease, chronic obstructive pulmonary disease, and myocardial infarct. Improvements in balance across covariates were measured using absolute values of standardized differences in the means or proportions of each covariate across the exposure groups, and were expressed as a percentage of the pooled SD. An absolute standardized difference <10%, which was also applied in our approach, is generally accepted as indicative of inconsequential residual bias.^7^

### Statistical methods

Continuous variables were presented as mean ± standard deviation for baseline characteristics and compared using Student’s *t*-test. Categorical variables are presented as frequencies with percentages (%) and were compared using the chi-square test or Fisher’s exact test. Cumulative incidences of primary endpoints were estimated by Kaplan–Meier survival curves and compared using log-rank tests. Univariate and multivariate regression analyses were used to adjust for differences in baseline variables between the two groups and to identify independent predictors of the primary endpoint. All analyses were two-tailed, and *p* values < .05 were considered statistically significant. All statistical analyses were performed using SPSS version 19 software (SPSS Inc., Chicago, IL, USA).

## Results

### Patients characteristics

Between January 1, 2008, and December 31, 2021, 1,319 patients were included, with 690 patients in the HD group and 629 patients in the PD group, according to the inclusion criteria. The baseline characteristics of the patients are summarized in Table 1.

**Table 1.** Patients characteristics.

	Before propensity-matching			After propensity-matching		
characteristics	HD (*n* = 690)	PD (*n* = 629)	*P*	HD (*n* = 461)	PD (*n* = 461)	*P*
Ethnicity (Han)	608 (88.1%)	553 (87.9%)	0.912	405 (87.9%)	406 (88.1%)	0.919
Age (years)	47.57 (17.22)	51.22 (16.55)	0.001	49.38 (16.91)	40.83 (16.40)	0.682
Male(%)	405 (58.7%)	318 (51.6%)	0.030	239 (51.8%)	245 (53.9%)	0.692
Weight (kg)	62.98 (9.53)	63.03 (9.27)	0.930	62.98 (9.68)	63.14 (9.31)	0.801
BMI (kg/m^2^)	25.96 (3.37)	25.50 (3.61)	0.018	25.64 (3.52)	25.71 (3.54)	0.770
Urine (mL)	227.26 (118.6)	256.25 (221.23)	0.036	237.26 (120.35)	255.25 (211.40)	0.056
PD (*n*, %)						
GN	267 (38.7%)	250 (39.7%)	0.846	187 (40.6%)	185 (40.1%)	0.919
DN	207 (30.0%)	194 (30.8%)		134 (29.1%)	139 (30.2%)	
HRD	136 (19.7%)	121 (19.2%)		85 (18.4%)	88 (19.1%)	
Others	80 (11.6%)	64 (10.2%)		55 (11.9%)	49 (10.6%)	
HB (g/L)	84.13 (20.59)	85.18 (20.33)	0.355	84.94 (10.53)	84.29 (21.09)	0.625
ALB (g/L)	28.99 (3.53)	29.05 (3.44)	0.798	28.99 (3.61)	28.93 (3.42)	0.782
NT-proBNP (pg/mL)	14877.88 (19608.3)	16641.38 (27405.3)	0.032	14987.88 (19647.2)	16470.38 (25401.7)	0.061
PTH (pg/mL)	689.53 (365.8)	709.23 (372.47)	0.333	704.76 (373.49)	693.63 (365.47)	0.648
GFR (mL/min)	7.97 (2.78)	8.59 (3.11)	0.034	8.62 (2.70)	8.58 (2.87)	0.792
Scr (umol/L)	761.02 (244.9)	736.81 (190.11)	0.019	751.02 (231.05)	739.94 (178.41)	0.058
BUN (mmol/L)	31.03 (9.50)	29.18 (7.18)	0.027	30.83 (8.60)	29.68 (7.20)	0.071
Comorbidities						
CAD	460 (66.7%)	485 (77.1%)	0.001	324 (70.3%)	319 (69.2%)	0.720
HF	496 (71.9%)	453 (72%)	0.957	334 (72.5%)	334 (72.5%)	1.000
DM	383 (55.5%)	360 (57.2%)	0.528	260 (56.4%)	257 (55.7%)	0.842
Cancer	47 (6.8%)	50 (7.9%)	0.429	11 (16.7%)	80 (17.4%)	0.793
HBP	378 (54.8%)	344 (54.7%)	0.973	255 (55.3%)	253 (54.9%)	0.895
COPD	97 (14.1%)	48 (7.6%)	0.001	39 (8.5%)	42 (9.1%)	0.727
PVD	61 (8.8%)	106 (16.9%)	0.001	51 (11.1%)	53 (11.5%)	0.835
MI	68 (9.9%)	94 (14.9%)	0.005	52 (11.3%)	53 (11.5%)	0.917

BMI, body mass index; PD, primary disease; GN, glomerulonephritis; DN, diabetes nephropathy; HRD, hypertensive renal disease; HB, hemoglobin; ALB, albumin; NT-proBNP, N-terminal pro brain natriuretic peptide; PTH, parathyroid hormone; GFR, glomerular filtration rate; Scr, Serum creatinine; BUN, blood urea nitrogen; CAD, coronary artery disease; HF, heart failure; DM, diabetes mellitus; HBP, high blood pressure; COPD, chronic obstructive pulmonary disease; PVD, peripheral vascular diseases; MI, myocardial infarction.

Before matching, the HD and PD groups included 690 (52.3%) and 629 (47.7%), respectively. As compared with the PD group, the HD group had younger patients (*p* = .001), a larger proportion of male patients (*p* = .030), higher BMI (*p* = .018), lesser urine output (*p* = .036), lower BNP (*p* = .032) and residual glomerular filtration rate (GFR) (*p* = .033), higher serum creatinine (*p* = .019) and urea nitrogen (*p* = .027), lower incidences of coronary heart disease (*p* = .001), peripheral vascular disease (*p* = .001) and myocardial infarction (*p* = .005); nevertheless, more patients in the HD group had COPD (*p* = .001).

Using a greedy matching algorithm, each patient in the PD group was matched in a 1:1 ratio to patients on PD with a caliper width of 0.2. After propensity matching, the population was restricted to 922 patients, i.e. 461 (50%) each in the HD and PD groups. After propensity score matching, there were no significant differences in baseline characteristics between the two groups (Table 1). The post-matching standardized differences for all 22 baseline covariates were <10%. The matched patients had a mean age of 49.38 ± 16.91 years, and 51.8% were men.

### Mortality rate in patients with ESRD

**Figure 1.**
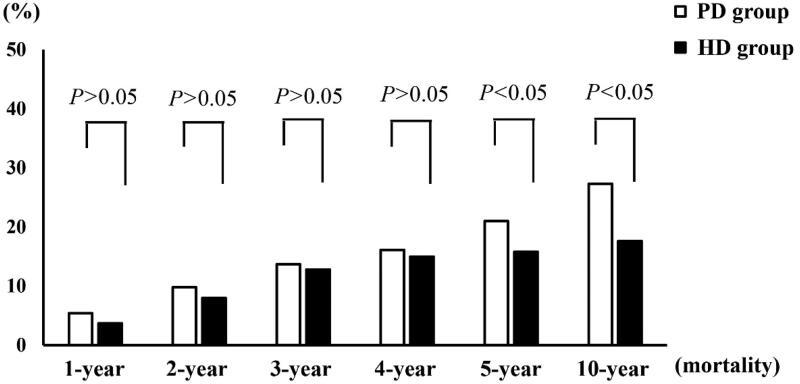
The mortality rate in patients with end-stage renal disease.

As shown in Figure 1, the 1-, 2-, 3-, and 4-year mortality rates of the matched patients were 3.7%, 8.0%, 12.8%, and 15.0% in the HD group and 5.4%, 9.8%, 13.7%, and 16.1% in the PD group, respectively. There were no significant differences in the 1-, 2-, 3-, and 4-year mortality rates between the HD and PD groups (all *p* > .05).

The 5- and 10-year mortality rates of the matched patients were 15.8% and 17.6% in the HD group and 21.0% and 27.3% in the PD group, respectively. The 5- and 10-year mortality rates in the HD group were significantly lower than those in the PD group (all *p* < .05).

### Results of comparison of survival rates between the two groups

**Figure 2.**
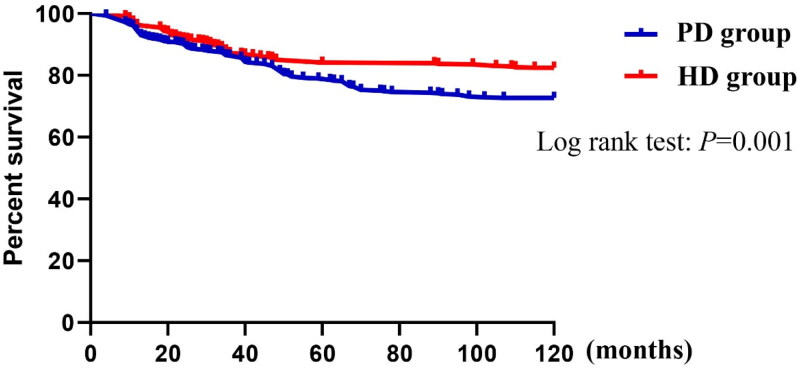
Kaplan–Meier curves showing survival of patients with end-stage renal disease. There was a significant difference between the PD and HD groups in terms of patient survival rate during follow-up. After a mean follow-up of 120 months, 82.4% of patients in the HD group and 72.7% in the PD group survived.

Kaplan–Meier curve analysis with the log-rank test was performed on the survival rates of the two groups of patients after matching. The results showed a significant difference in the survival rates between the two groups (*p* = .001) (Figure 2).

### Factors associated with all-cause death in ESRD

While survival outcome was the dependent variable, ethnicity, sex, age, weight, urine output, BMI, primary disease, hemoglobin, albumin, NT-proBNP, PTH, residual renal function, serum creatinine, blood urea nitrogen, coronary artery disease, heart failure, PVD, diabetes mellitus, hypertension, malignant neoplastic diseases and chronic obstructive pulmonary disease were independent variables. Logistic multifactor regression analysis performed on the basic data of the two groups of patients revealed that age, weight, hypertension, serum creatinine, and combined neoplasm influenced the survival rate of patients (*p* < .05) (Table 2).

**Table 2.** Multivariate analysis for factors associated with all-cause death in ESRD.

Variables	Adjusted OR for all patients (95% CI)	P value	Variables	Adjusted OR for HD (95% CI)	P value	Variables	Adjusted OR for PD (95% CI)	P value
Age	1.018 (1.008–1.028)	0.001	Age	1.018 (1.002–1.035)	0.030	Age	1.015 (1.002–1.028)	0.021
Weight	0.966 (0.949–0.983)	0.001	HBP	1.994 (1.140–3.489)	0.016	Weight	0.947 (0.925–0.970)	0.001
HBP	1.489 (1.060–2.090)	0.022	PTH	1.001 (1.000–1.002)	0.005			
Scr	0.996 (0.990–1.000)	0.047	Scr	0.999 (0.998–1.000)	0.054			
Cancer	1.838 (1.233–2.741)	0.003	PVD	2.844 (1.424–5.685)	0.003			

HRD, hypertensive renal disease; Scr, Serum creatinine; PTH, parathyroid hormone; PVD, peripheral vascular disease.

### Analysis of factors influencing the survival rate of patients in the HD group

Logistic multifactor regression analysis of the basic data of patients in the HD group revealed that age, hypertension, PTH, serum creatinine, and PVD were factors that influenced the survival rate of patients (*p* < .05) (Table 2).

### Analysis of factors influencing the survival rate of patients in the PD group

Logistic multifactor regression analysis of the basic data of patients in the PD group demonstrated that age and weight influenced the survival rate of patients (Table 2).

## Discussion

Currently, the treatment for ESRD consists of HD, PD, and kidney transplantation. Owing to the shortage of kidney sources and other factors, HD or PD is the primary treatment for managing patients with ESRD.^8^ In recent years, with the continuous development of medical technology, PD and HD have significantly improved the survival rates of patients with ERSD. Studies from China have demonstrated the advantages and disadvantages of the two dialysis modalities; however, these studies were mostly limited to small sample sizes or had short-term follow-ups, and the results were inconsistent. For example, Herrera et al. concluded that the important difference between HD and PD was that the survival rate of patients on HD was higher than that of patients on PD, which was significant after 5 years of follow-up in 12,508 patients undergoing dialysis.^9^ Similarly, Mukhopadhyay et al. concluded that the survival rate of patients on HD was higher than that of patients on PD after 5 years of follow-up in 130,324 patients undergoing dialysis.^7^ Furthermore, in a study of patients on maintenance dialysis with a 5-year follow-up, Yao X et al. noted that the survival rates of patients on PD were better than of those undergoing HD, and these results were more applicable in the Chinese population.^10^ Additionally, factors other than HD modality influenced the survival of patients with ESRD. To further clarify the difference of survival rates between the two treatment modalities and related influencing factors, we analyzed the clinic data of patients undergoing HD and PD with long-term follow-up in our hospital to provide a theoretical basis for clinicians to select treatment modalities, take effective preventive measures, and improve survival prognosis, while accounting for the medical conditions in our region.

Of the 1,319 patients who met the inclusion and exclusion criteria for this study, 690 were undergoing HD and 629 were undergoing PD. Statistical analysis of the baseline data revealed that age, sex, weight, BMI, urine output, NT-proBNP, residual renal function, serum creatinine, blood urea nitrogen, concomitant coronary artery disease, COPD, PVD, and MI were significantly different between the two groups. To reduce the impact of an imbalance in baseline information on survival, propensity score matching was applied, and 461 matched pairs of patients (922 patients) were finally matched, with no significant difference in baseline data between the two groups.

Using the 10-year follow-up of the 922 matched patients, we found that there was no significant difference between the 1-, 2-, 3-, and 4-year mortality rates of patients in the HD and PD groups, indicating that both renal replacement therapy modalities had the same impact on survival in the short term, which depended not only on the development of renal replacement, but also on the regular and effective follow-up in the early stage and timely adjustment of treatment regimens by physician. Wong B et al. concluded after a follow-up of 2,032 patients undergoing dialysis that there is no difference in short-term survival between the two treatment modalities of HD and PD.^11^ Continued follow-up of the patients for 5 and 10 years revealed that the mortality rate in the HD group was significantly lower than that in the PD group. Further log-rank test analysis of the Kaplan–Meier survival curves for both groups revealed that with the extension of follow-up time, especially after 5–10 years, the survival rate was higher in the HD group than that in the PD group. This is related to the increase in age at dialysis and the gradual increase in dialysis-related complications in patients with ESRD as the duration of dialysis increases, which affects patient survival. In patients undergoing PD, the peritoneum is stimulated by blood glucose for a long time, and peritoneal sclerosis decreases ultrafiltration, ability to remove toxins, and adequacy of dialysis, amplifying the decrease in survival rate in patients undergoing PD as compared to those undergoing HD. Kim H et al. showed that the overall mortality rate of patients on PD was higher than that of patients undergoing HD in a 5-year follow-up study of 7,049 propensity score-matched pairs of patients undergoing dialysis. The survival rate in the PD group was lower than that for patients on HD, which is consistent with the findings of our study.^12^ In contrast, a MATA analysis of 14 randomized controlled trials by Lu et al. showed no significant difference in 1-year survival between patients on PD and HD, both dialysis modalities reduced complications and improved the nutritional status of patients and their quality of life.^13^ However, they did not conduct further studies on the long-term survival rate of both groups. To understand the factors influencing the survival rate of patients treated with dialysis for ESRD, a regression analysis of the influencing factors was performed. The independent risk factors affecting the survival prognosis of patients on maintenance dialysis included the following (1) age: compared to younger individuals, older individuals have a higher risk of death and a lower survival rate, probably because older patients have more underlying diseases and are prone to combined multiple organ functions and metabolic decompensation, making them more susceptible to complications such as heart rate, blood pressure, and electrolyte disorders. Moreover, when the role of older adults in the family and society becomes less important, they are prone to the influence of psychological factors, and their ability to respond to stress decreases during dialysis, leading to increased mortality. Moreover, as the duration of dialysis increases, dialysis-related complications increase gradually, and the patients are at an increased risk of death. This is consistent with several relevant studies from countries other than China, such as the study by Weinhandl et al. which divided patients gradually maintenance dialysis by age into two subgroups i.e. older than 65 years and younger than 65 years. The survival rate analysis found that the survival rate of patients older than 65 years was significantly lower than that of patients younger than 65 years, which also elucidated the effect of age on the survival rate of patients on dialysis.^4^ (2) Weight: The mortality rate of patients on maintenance dialysis was negatively correlated with the pre-dialysis weight course. the lower the weight, the higher the mortality rate. Low body weight indicates that the patients in the pre-dialysis setting are weaker and malnourished, which may be related to decreased appetite, poor digestive function, and inadequate nutritional intake before dialysis. Patients not gradually dialysis are more likely to take multiple drugs to remove toxins, correct electrolyte disorders, and rescue complications to delay entry into the dialysis phase, which is more likely to lead to gastrointestinal dysfunction and weight loss. Therefore, low weight before dialysis increases the risk of patient death.^14^ Most patients on maintenance dialysis have inadequate dialysis and substandard dry weight. They are significantly overweight, mostly due to inadequate fluid clearance and fluid overload, increased risk of cardiovascular disease, and increased risk of death.^15^(3) Hypertension: the higher the blood pressure of the patient before dialysis, the greater the risk of death. High blood pressure before dialysis directly reflects the obvious loss of renal function, excessive fluid load, and poor blood pressure control. The higher the blood pressure, the higher the likelihood of heart failure, coronary heart disease, stroke, and other cardiovascular and cerebrovascular events, to increase the risk of death for patients. Several studies have shown that hypertension seriously affects the quality of life and long-term survival of patients, leading to a significantly increased risk of death from cardiovascular and cerebrovascular diseases.^16^ (4) Serum creatinine levels: The higher the pre-dialysis blood creatinine level, the lower the survival rate of patients. Blood creatinine is a direct response to renal function and represents the ability of the kidneys to remove toxins. High pre-dialysis blood creatinine means that patients have a significant decline in residual renal function, delayed to dialysis, poor drug treatment, and patients mostly have complications such as volume overload, heart failure, hypertension, severe anemia, nutritional deficiency, and bone disease. Consequently, they have a decreased survival rate. (5) The presence of oncologic disease before dialysis is an influential factor in the survival rate of patients with ESRD. Neoplastic diseases are chronic progressive diseases that threaten the life of patients. If patients have tumors before dialysis, the final cause of death is mostly tumor-related. Patients with ESRD have low immunity and poor nutritional status, coupled with limited use of surgical or chemotherapeutic drugs and treatment methods, mostly because the renal function is affected. Patients with poor prognoses have decreased survival rates. Ali et al. pointed out that the presence of concomitant neoplastic disease in patients on maintenance dialysis had a negative influence on survival, and the causes of death in patients were mostly tumor-related, which was consistent with our findings.^17^ We analyzed the factors influencing the survival rate of patients on HD. In addition to age, hypertension, and serum creatinine, the factors influencing the survival rate of patients on HD included PTH and PVD. Elevated PTH levels are caused by low calcium and high phosphorus levels induced by GFR in patients with ESRD, which increases the secretion of secondary PTH, which is the underlying etiology of renal osteodystrophy. Elevated PTH not only represents chronic kidney disease at the end stage but also signals poor dietary management and disturbed nutritional status in patients, who mostly have abnormal calcification of the cardiovascular system and abnormal bone metabolism, and these are independent risk factors for death in patients on dialysis. Nasri H et al. concluded from a study of 73 patients on dialysis that structural and functional cardiac indicators in patients on maintenance fluid dialysis were significantly correlated with serum calcium, phosphorus, and PTH levels. Secondary hyperparathyroidism is an influential factor in left ventricular hypertrophy and reduced left ventricular ejection fraction in patients on maintenance dialysis. Close attention should be paid to PTH control to reduce the risk of cardiovascular disease and death.^18^ The presence of PVD before HD affects the survival rate of patients undergoing HD, including those with peripheral arterial and venous diseases. It is also a manifestation of systemic vascular disease and predicts an increased incidence of cardiovascular and cerebrovascular diseases. The impact on survival among patients on HD is particularly important, as it affects the establishment of access in patients on dialysis and the maintenance of dialysis adequacy in later stages. A study by Lo et al. reported that peripheral vascular disease increased the all-cause mortality and incidence of coronary heart disease in patients undergoing maintenance dialysis, and that aggressive treatment could prolong the survival of patients on dialysis.^19^ Logistic risk regression analysis for patients on PD found that age and weight are influential factors in the survival of patients on PD. age is an important influencing factor in the maintenance dialysis treatment of patients with ESRD, and survival rate of patients undergoing HD and PD decreased significantly with older age. He Z et al. studied 937 patients undergoing dialysis who were followed up for 5 years and found that PD treatment was a more desirable option for younger patients with ESRD, indicating that age is an influencing factor in patients on PD.^20^ Furthermore, body weight is an important nutritional indicator prior to dialysis, and it is an important indicator of dialysis adequacy. Further, good nutritional status is beneficial in reducing mortality in patients on maintenance dialysis.^21^^,^^22^

### Limitations

The present study has some limitations. First, the results were obtained through an observational study. Despite using a multivariate analysis, the possibility of unmeasured or residual confounding factors cannot be excluded. Second, our study had a single-center design with a limited sample size. Third, we did not observe dynamic changes in the dialysis status, related complications, or comorbidities during follow-up. Studies have suggested that patient outcomes may be related to the dynamic changes in these factors. Further studies that include a larger sample size and continuous dynamic observation of clinical data would be needed to improve the generalizability of study findings.

## Conclusion

This study found that the long-term mortality rates were higher in the PD group than that in the HD group, indicating that the HD strategy is superior to the PD strategy. HD may be an effective dialysis modality in future clinical practice for physicians to choose, thereby improving the survival rate of end-stage kidney disease patients.
